# Simultaneous pancreas and kidney transplantation for end-stage kidney disease patients with type 2 diabetes mellitus: a systematic review and meta-analysis

**DOI:** 10.1007/s00423-021-02249-y

**Published:** 2021-07-19

**Authors:** Yu Cao, Xiaoli Liu, Xiangyu Lan, Kaiwen Ni, Lin Li, Yingxin Fu

**Affiliations:** 1 Department of Kidney and Pancreas Transplantation, Tianjin First Central Hospital, Tianjin, China; 2 Research Center of Clinical Epidemiology, Peking University Third Hospital, Beijing, China

**Keywords:** Simultaneous pancreas and kidney transplantation, Kidney transplantation alone, Type II diabetes, Survival outcomes, Meta-analysis

## Abstract

**Purpose:**

The indications for patients with type 2 diabetes mellitus (T2DM) combined with end-stage kidney disease (ESKD) undertaking simultaneous pancreas and kidney transplantation (SPK) remain an unresolved issue. This study aimed to systematically review the survival outcomes of SPK among T2DM-ESKD patients.

**Methods:**

Online databases including PubMed, MEDLINE, EMBASE, and the CENTRAL Library, CNKI, Chinese Biomedical Literature Database, and Wan-Fang database were used to locate the studies of ESKD patients with T2DM undertaking SPK up to May 2021. A third reviewer was consulted if there were disagreements. Data were analyzed with STATA (15.0).

**Results:**

Nine cohort studies were identified. The pooled 1-year, 3-year, and 5-year patient survival rates of patients with T2DM and ESKD after SPK were 98%, 95%, and 91% respectively. Comparing the treatment effect of SPK between type 1 diabetes mellitus (T1DM) and T2DM, the survival estimates were comparable. For T2DM patients, SPK had a survival advantage compared with KTA.

**Conclusions:**

The synthesized clinical outcomes of T2DM patients with ESKD after SPK were relatively better than KTA, but a subset of T2DM-ESKD patients who would benefit the most from SPK was to be defined.

PROSPERO registration number

CRD42019118321. Date of registration: 14 Jan 2019 (retrospectively registered)

**Supplementary Information:**

The online version contains supplementary material available at 10.1007/s00423-021-02249-y.

## Introduction

It was estimated that there were more than 463 million people were living with diabetes mellitus (DM) worldwide, and more than 90% of them were diagnosed with type 2 diabetes (T2DM) [1, 2]. In Europe, the number of DM is estimated to be 58 million [2]. Over the past years, China has witnessed a surging prevalence of diabetes, with the largest number of diabetic patients in the world [3] and ranked number one in the 2019 International Diabetes Federation Diabetes Atlas Report [2]. Furthermore, diabetes is the leading cause of end-stage kidney disease worldwide; in conjunction with hypertension, it resulted in at least 80% end-stage kidney disease (ESKD) [4]. In the USA, Japan, New Zealand, and Singapore, about 50% of ESKD are primarily due to DM [4].

Since the first pancreas transplantation was done at Minnesota University in 1966, with the improvement of surgical techniques and introduction of immunosuppressive agents of cyclosporine, the number of pancreas transplantation has increased steadily, especially for simultaneous pancreas transplantation [5, 6]. SPK has been a medically effective and cost-effective method for T1DM, but there was no consensus on SPK for the T2DM population, especially in the aspect of selection criteria [6, 7]. In the 2020 Kidney Disease: Improving Global Outcomes (KDIGO) guideline, patients with ESKD and T1DM were recommended for SPK, while there were no suggestions for those with T2DM [8]. Data on SPK outcomes in T2DM patients began appearing in the annual International Pancreas Transplant Registry (IPTR) reports since the mid-1990s [9, 10]. The number has gradually increased with treatment outcomes equivalent to or better than other treatment alternatives on T2DM nephrological patients [11]. The cases of SPK were steadily increasing in Europe as well [12]. Considering the growing size of T2DM-ESKD recipients receiving SPK and the need to synthesize existing knowledge to inform clinical practice, we sought to review systematically and summarize available survival data in these patients. We planned to (i) synthesize the risk of death after SPK for T2DM-ESKD patients; (ii) assess the quality of available epidemiological data; (iii) summarize the hazard risk of mortality between SPK T2DM recipients and their counterparts; and (iv) estimate the relative risk of commonly reported complications between SPK T2DM recipients and their counterparts.

## Methods

This meta-analysis was written in accordance with Preferred Reporting Items for Systematic Reviews and Meta-Analyses [13].

### Eligibility criteria

This review included cohort studies estimating the survival outcome of SPK for T2DM patients combined with ESKD since no trials were available. All studies that reported SPK survival outcomes of T2DM-ESKD patients in English or Chinese were included. There were no restrictions on the type of setting. The year of publication was limited for Chinese studies. Those conducted before 2010 were excluded during the study selection process, considering the implementation of the Donation after Citizen’s Death in 2010 [14]. Primary outcomes were patients’ and grafts’ survival rates. Secondary outcomes were hazard ratio between T1DM and T2DM, SPK, and KTA, and risk ratio of complications was recorded as well.

### Information sources, search strategy, and records management

Only quantitative studies were searched. PubMed, MEDLINE (1946 onwards), EMBASE (1947 onwards), the CENTRAL trials registry of the Cochrane Collaboration (1948 onwards), China National Knowledge Infrastructure (CNKI, 1994 onwards), Chinese Biomedical Literature Database (CMB, 1978 onwards), and Wan-Fang database (1998 onwards) were searched to May 2021. The specific search strategies were created by two team members in consultation with an expert in medical informatics. Search strategies were included in Supplemental digital contents Table 1 (SDC-Table S1). As relevant studies were identified, the reviewers checked for additional relevant articles. Records identified through the database were managed with NoteExpress, which is an information manager for researchers and designed to help organize research notes and bibliographic references and generate bibliographies automatically (http://www.inoteexpress.com/aegean/).

**Table 1:** Characteristics of included studies

First author, year, country	Study design	Data sources	No. cases	Study period	Mean/median age (yr)	Sex (M%)	BMI (kg/cm^2^)	Duration of DM (yr)	Follow-up	Meta-analysis
Sampaio [15], 2011, USA	Cohort study	UNOS	SPK1: 6141 SPK2: 582	2000–2007	SPK1: 40 (34–46)^**^ SPK2: 47 (40–52)	SPK1: 61^**^ SPK2: 68.7	SPK1: SPK2^**^ < 18.5: 2.8/2.8 18.5–25: 54.2/43.9 25–30: 32/36.2 > 30: 10.7/17.1	SPK1: 26 (21–32)^**^ SPK2: 21 (16–27)	Up to 7 y	Meta-B Meta-C
Margreiter [16], 2013, Austria	Cohort study	Innsbruck Medical University	SPK1: 195 SPK2: 21 KTA2: 32	2002/1–2009/9	SPK1: 41.6 ± 9.2^**^ SPK2: 53.6 ± 5.9 KTA2: 63.5 ± 5.6^@@^	SPK1: 66.7^**^ SPK2: 8 KTA2: 90.6^@@^	SPK1: 23.4 ± 3.1^**^ SPK2: 25.1 ± 3.3 KTA2: 26.6 ± 2.5^@^	SPK1: 24 ± 11.2** SPK2: 15.8 ± 6.3 KTA2: 15.1 ± 7.9^@@^	Mean: 7.3 y	Meta-A Meta-B Meta-C
Jeon [17], 2016, South Korea	Cohort study	Seoul National University Hospital and Samsung Medical Center	SPK1: 20 SPK2: 28 KTA1: 3 KTA2: 69	2000/2–2011/12	SPK1: 34.6 ± 5.8^**^ SPK2: 47.3 ± 9.9 KTA2: 53.6 ± 9	SPK: 54.2^*^ KTA: 72.2	SPK: 22.9 ± 4^*^ KTA: 24.1 ± 3.2	SPK: 18.3 ± 5.5^**^ KTA: 16.4 ± 7.1	Mean: 53.4 m	Meta-A Meta-B
Gruessner [11], 2017, USA	Cohort study	UNOS	SPT2: 1322	1995–2015	SPT2: 46.4 ± 8.3	70.8	< 18.5: 24 18.5–24.9: 535 25–29.9: 540 ≥ 30: 44	SPT2: 21.1 ± 7.9	Up to 20 y	Meta-A (only for survival rate pancreas graft)
Fu [18], 2017, China	Cohort study	Tianjin First Central Hospital	SPK1: 36 SPK2: 73	2008–2016	SPK1: 35.4 ± 8.4^*^ SPK2: 50 ± 8	SPK1: 58.3^*^ SPK2: 82.2	SPK1: 21.5 ± 3.5^*^ SPK2: 23.9 ± 3.3	SPK1: 17.6 ± 5.4^*^ SPK2: 20.8 ± 6.7	NR	Meta-B
Gondolesi [19], 2018, Argentina	Cohort study	Fundación Favaloro University Hospital	SPK1: 35 SPK2: 10	2008/4–2016/3	SPK1: 36.9 ± 7.8^*^ SPK2: 53.7 ± 7.3	54.3	SPK1: 23.1 ± 3.5^**^ SPK2: 25.72	SPK1: 24.3 ± 6 .5^*^ SPK2: 20.4 ± 6.5	Up to 8 y	Meta-A Meta-B
Alhamad[26], 2019, USA	Cohort study	UNOS	SPK2: 669 KTA2: 25,403	2000/1–2016/12	SPK,P + /SPK,P − /KAT2^***^: Age group	SPK,P + /SPK,P − /KAT2/LDT2^***^: 71.7/80/66.5/71	SPK,P + /SPK,P − /KAT2^***^: BMI group	NR	Up to 18 y	Meta-A (for survival rate of patient and kidney graft) Meta-C
Hau[28], 2020, Germany	Cohort study	University Hospital of Leipzig	SPK1: 89 SPK2: 12 KTA2: 26	2001–2013	SPK1/SPK2/KTA2: 42.3 ± 8.4/48.7 ± 10.6/ 61.5 ± 8.6^@@^	SPK1/SPK2/KTA2: 55.1/66.7/80.8	SPK1/SPK2/KTA2: 24.8 ± 4.1/ 26.4 ± 4.9/ 28.6 ± 3.1^@^	SPK1/SPK2/KTA2: 27.6 ± 7.9/18.7 ± 9.8/ 18.9 ± 8.9	5 y	Meta-A Meta-B
Fu[27], 2021, China	Cohort study	Tianjin First Central Hospital	KTA2: 85 SPK2: 71	2015–2020	KTA2: 47.6 ± 11.4 SPK2: 48.8 ± 7.8	KTA2: 80 SPK2: 90	KTA2: 25.5 ± 4 SPK2: 24.6 ± 3	KTA2: 12.4 ± 7.2 SPK2: 16 ± 5.7	Median: 2 y	Meta-A Meta-C

Note: *UNOS*, United Network for Organ Sharing; *SPK1*, T1DM recipients with SPK; *SPK2*, T2DM recipients with SPK; *KTA1*, T1DM recipients undertaking deceased kidney transplantation alone; *Cp*, C-peptide level (ng/mL); *NR*, not reported

^*^There was no statistical significance between SPK1 and SPK2

^**^There was statistically significant between SPK1 and SPK2

^@^There was no statistically significant between SPK2 and KTA2

^@@^There was statistical significant between SPK2 and KTA2

^***^P < 0.05 for chi-squared tests comparing differences between SPK,P + , SPK,P − , DD-KA, and LD-KA groups; Meta-A: meta-analysis of synthesizing survival outcome of T2MD after SPK. Meta-B: meta-analysis of summarizing hazard ratio between T1DM and T2DM after SPK, Meta-C: meta-analysis of synthesizing hazard ratio between SPK and KTA among T2DM)

### Study selection

The titles and abstracts of all of the references generated by search strategies were screened independently by two review members to identify eligible studies. Disagreements were resolved by discussion and consensus between the two reviewers. If disagreement persisted, the final decision was made by consensus with the involvement of the third member of the team. The full-text articles of included abstracts and uncertain abstracts were retrieved and reviewed by two members for inclusion separately. Reasons for study exclusion were recorded.

### Data collection process

Two authors independently extracted and record data based on a standardized data extraction form (EXCEL form) designed by YXF and YC. The following items were extracted from the identified articles, name of the first author, publication year, title, study purpose, country, city/region, data source, study design, definition of T2DM, operation technique, number of cases, study period, age, BMI, sex, duration of DM, pre-operation comorbidities, induction agents, immunosuppressive agents, follow-up period, definitions and rates of complications, definition of graft failure, survival rate, and HR. Adjusted data were preferentially selected if available [20]. When the eligible studies failed to provide specific survival data and HR with 95% CI, the data in figures were extracted using Engauge Digitizer (version 10.11 http://markummitchell.github.io/engauge-digitizer/), a free publicly available software, and the HR with 95%CI was calculated using methods suggested by Tierney et al. [21].

### Risk of bias in individual studies

The Newcastle–Ottawa Scales (NOS) recommended by the Cochrane handbook were adopted for quality assessment [22, 23]. Giving that enrolled studies were retrospective cohort design with various sources of heterogeneity, modification on NOS was undertaken. The final customized NOS was presented in SDC-Table S2. Two reviewers independently appraised the study quality. Disagreements between the reviewers over the risk of bias were resolved by discussions with a third reviewer (YXF).

### Data synthesis

Survival rates and HRs were combined with the random effect model. All statistical syntheses and analyses were performed using STATA (15.0). Statistical heterogeneity was assessed using the I^2^ statistic (< 0%: very slight heterogeneity; 30% to 60%: may represent moderate heterogeneity; 50% to 90%: may represent substantial heterogeneity; 75% to 100%: considerable heterogeneity) [24]. If the heterogeneity was above 50%, sensitivity analysis would be conducted. Evidence surrounding definitions of DM, baseline characteristics of recipients, definitions of graft failure, and complications which might play a role in survival outcomes was synthesized qualitatively. Funnel plots were not used to visualize the publication bias in each group since the number of included studies in the meta-analysis was less than 10 [22, 25].

## Results

### Search results

**Fig. 1:**
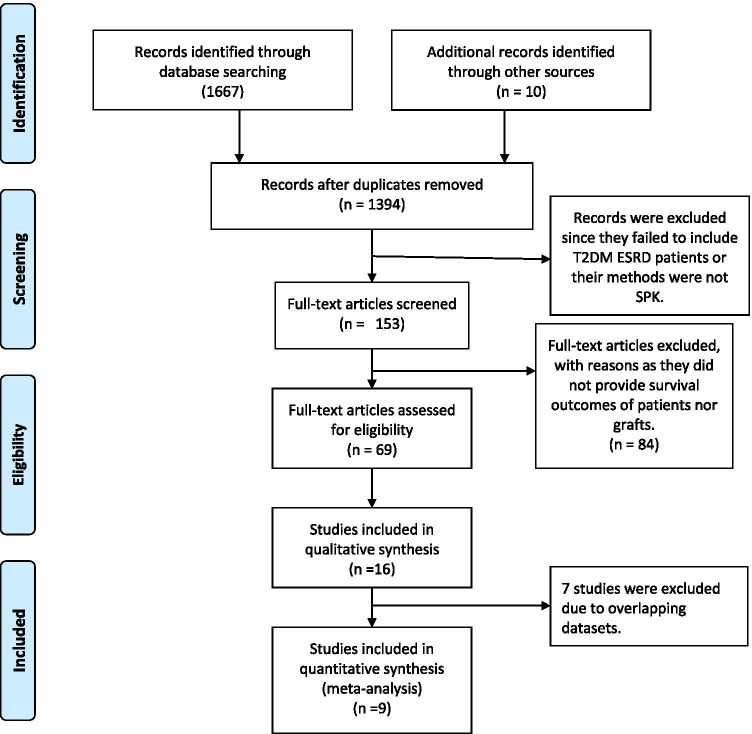
PRISMA flowchart

The search yielded abstracts for 1677 publications. After excluding duplicate articles and screening the abstracts, 1394 were remained for further review. Then, 153 copies of the full published version of each study were obtained, after excluding records which did not refer to SPK among recipients with T2DM in the title or abstract. Sixty-nine full texts were excluded next due to lack of survival outcomes of patients or grafts, leaving 16 eligible publications. Next, among the 16 studies, 9 were involved in the difference data synthesis process [11, 15–19, 26–28]. Considering the study quality and data completeness on primary outcomes, 6 were included for synthesizing survival outcomes of T2MD patients after SPK [11, 15–17, 19, 26–28], 6 studies were included for summarizing the hazard ratio between T1DM and T2DM after SPK [15–19, 28], 3 studies were included for synthesizing hazard ratio between the SPK group and the KTA group [16, 26, 28], and each meta-analysis had no overlapping samples. (Fig. 1, Table 1). The details of excluded studies with overlapping samples were in the SDC-Table S3.

### Study characteristics

Characteristics of the included studies were presented in Table 1 and Table 2 (additional information in SDC-Table S4). All included studies were retrospective cohort studies. In accordance with the eligible criteria, all reports included a cohort of T2DM ESKD patients undertaking SPK. Considering the geographical coverage of the study, included studies were from the USA, Argentina, Germany, Austria, South Korea, and China.

**Table 2:** Case definition of included studies

First author, year, country	Study design	Data sources	Definition of diabetes mellitus		Operation description	Definition of renal failure	Definition of pancreas failure
			T2DM	T1DM			
Sampaio[15], 2011, USA	Cohort study	OPTN/UNOS	Diabetes type definition was taken from the SPK transplant recipient registration form and was based on the diagnosis of ESPD (code 5001 and 5002, corresponding to T1DM and T2DM, respectively)		NR	KRT; RTD; PD; EFU;	PD; pancreas failure
Margreiter[16], 2013, Austria	Cohort study	Innsbruck Medical University	Fasting C-peptide was used as the main criterion for defining T2DM, and the absence of autoantibodies (against islet cells, insulin, glutamic acid decarboxylase, or tyrosine phosphatase IA2)	T1DM was defined as an early-onset disease with a sudden need for insulin, presence of one or more autoantibodies, and C-peptide negativity	Y	KRT; RTD; PD	NR
Jeon[17], 2016, South Korea	Cohort study	Seoul National University Hospital and Samsung Medical Center	Patients who did not meet the diagnostic criteria of type 1 DM were categorized to type 2 DM. Those who had a low cardiovascular risk and a body mass index (BMI) of < 30 kg/m^2^ among type 2 DMESKD patients were considered acceptable for SPK	Age at disease onset < 40 yr, and the maximum weight ≤ 105% of the standard body weight with at least one of the following: (i) history of diabetic ketoacidosis, (ii) starting insulin therapy within one yr after diagnosis. The laboratory results of low fasting C-peptide (< 0.8 ng/mL) and presence of anti-pancreatic or anti-insulin autoantibodies supported the classification of type 1 diabetes	NR	KRT; RTD; PD	PD; insulin resumption; taking oral hypoglycemic agents due to hyperglycemia
Fu[18], 2017, China	Cohort study	Tianjin First Central Hospital	NR	NR	Y	KRT; RTD; PD	NR
Gruessner[11], 2017, US	Cohort study	OPTN/UNOS	The guidelines of the American Diabetes Association (ADA)		NR	KRT; RTD; PD	PD; insulin resumption
Gondolesi[19], 2018, Argentina	Cohort study	Fundación Favaloro University Hospital	T2DM: were patients usually diagnosed over 30 years of age, they often present with associated increased body weight; high blood pressure; and/or cholesterol levels. Initial management has usually been diet. Although C-peptide is not considered diagnostic of T2DM, it usually divides patients as resistant to insulin (when its value is greater than 2.35 ng/mL) or as the absence of insulin production, when the value is normal or below normal. Determinations of antibodies should have been negative in this group of patients	T1DM: Often diagnosed in childhood, not associated with excess body weight, often associated with higher-than-normal ketone levels at diagnosis, treatment should have been done with insulin. And, patients should have test positive for autoantibodies (GAD, IA2, or ZnTA8), although there have been described cases with negative autoimmunity	Y	Serum creatinine levels and creatinine clearance	Measuring C-peptide, serum glycemia, insulin levels
Alhamad[26], 2019, USA	Cohort study	OPTN/UNOS	Diabetes type definition was taken from the SPK transplant recipient registration form and was based on the diagnosis of ESPD (code 5001 and 5002, corresponding to T1DM and T2DM, respectively)		NR	KTR; RTD; PD	UNOS definition based on program-specific reports
Hau[28], 2020, Germany	Cohort study	University Hospital of Leipzig	The guidelines of the American Diabetes Association (ADA) and the World Health Organization (WHO). Selection criteria for pancreas transplantation in T2DM in our center include patients < 60 years with a body mass index of < 30 kg/m^2^, fasting C-peptide levels < 10 ng/mL, insulin requirement for a minimum of 5 years with daily requirements of less than 1 U/kg per day, absence of pancreatic antibodies (anti-glutamic acid decarboxylase (GAD)), islet cell antibodies (ICA), anti-tyrosine phosphatase (anti-IA2), absence of severe vascular disease and adequate cardiac function		Y	KRT; RTD; PD; EFU	PD; pancreas failure with resumed insulin therapy; EFU
Fu[27], 2021, China	Cohort study	Tianjin First Central Hospital	1999 WHO guidelines and 2013 Guidelines for the prevention and control of type 2 diabetes in China		Y	KRT; RTD; PD	PD; PT; resumption of the daily scheduled insulin

*KRT*, kidney retransplantation; *RTD*, return to dialysis; *PD*, patient death; *EFU*, end of follow-up; *Cp*, C-peptide level (ng/mL); *BMI*, body mass index; *FPG*, fasting plasma glucose; *GDM*, gestational diabetes mellitus; HDL-*C*, high-density lipoprotein cholesterol; *OGTT*, oral glucose tolerance test; *PG*, plasma glucose; *TG*, triglycerides; *HbA1c*, glycosylated hemoglobin. Blood glucose rather than A1C should be used to diagnose the acute onset of type 1 diabetes in individuals with symptoms of hyperglycemia and inform the relatives of patients with type 1 diabetes of the opportunity to be tested for type 1 diabetes risk, but only in the setting of a clinical research study; *Y*, yes; *NR*, not reported

### Definition of T2DM and definition of renal failure and pancreas failure

As shown in Table 2, a variety of DM definitions has been witnessed among the included studies. Studies adopted center-specific criteria in selecting DM candidates for SPK [16, 17, 26–28], which classified DM around the C-peptide level, BMI of 30 kg/m^2^, age, and pancreatic antibodies [15–17, 19, 29–31]. UNOS’s definition of T2DM was based on the SPK transplant recipient registration form and the diagnosis of end-stage pancreas disease (ESPD) [15].

A consensus on the definition of renal failure among enrolled studies was witnessed, which was kidney retransplantation, returning to dialysis, or patient death. However, the definitions of pancreas graft failure varied. Most studies defined pancreas graft failure as insulin resumption, patient death, or pancreas graft removal.

### Methodological quality of included studies

Methodological quality scores ranged from 4 to 8 on a modified scale of 0 to 11 (Table 3). A majority of studies showed good quality in patient selection and outcome assessment [11, 15–19, 26, 28]. The main heterogeneity between studies might arise from the sample size disparity, poor comparability of cohorts on the basis of study design or analysis, and insufficient reporting of follow-ups. Specifically, most studies [16–19, 27, 28] had a sample size of T2DM undertaking SPK below 100 and the number in UNOS studies [11, 15, 26] was more than 500; only 3 studies [15, 26, 27] reported adjusted hazard ratio on T1DM vs. T2DM or SPK vs. KTA; the majority of studies neither reported median or mean follow-up period, nor described the details of the follow-up-losses (Table 1 and 3) [11, 15,19, 26,28].

**Table 3:** Quality assessment: modified Newcastle–Ottawa scale for cohort studies (11 stars total)

Author, year	Selection				Comparability	Outcome			Total stars
	S1	S2	S3	S4	C1	O1	O2	O3	
Sampaio[15], 2011	**	*	*		*	*	—	—	6/11
Margreiter[16], 2013		*	*		—	*	**	—	5/11
Jeon[17], 2016	**	*	*	*	—	*	*	—	7/11
Fu[18], 2017	*	*	*	*	—	—	—	—	4/11
Gruessner[11], 2017	**	NA	*	*	NA	*	*	—	6/9
Gondolesi[19], 2018		*	*	*	—	*	—	—	4/11
Alhamad[26], 2019	**	*	*	*	*	*	*	—	8/11
Hau[28], 2020		*	*	*	—	*	**	—	6/11
Fu[27], 2021	*	*	*	*	*	*	—	*	7/11

More stars (*) indicate higher quality of the study. S1, representativeness of exposed cohort; S2, selection of nonexposed cohort; S3, ascertainment of exposure; S4, study was published within 5 years (after 2016); C1, comparability of the cohort on basis of design or analysis; O1, assessment of outcome; O2, was follow-up long enough for outcomes to occur ; O3, adequacy of follow-up; *NA*, not applicable; For details, please refer to SDC-Table S2.

### Survival rates

#### Pooled 1-year, 3-year, and 5-year survival rates of patients, kidney graft, and pancreas graft (Fig. 2A–C)

**Fig. 2:**
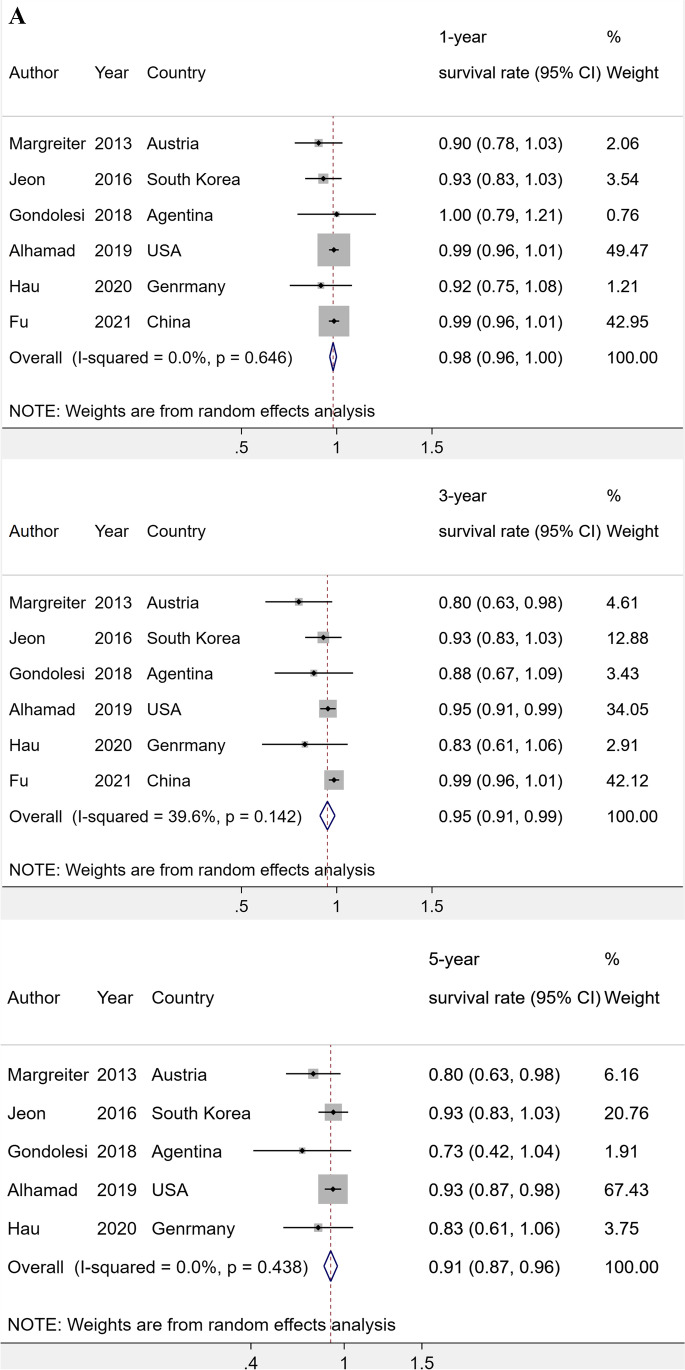

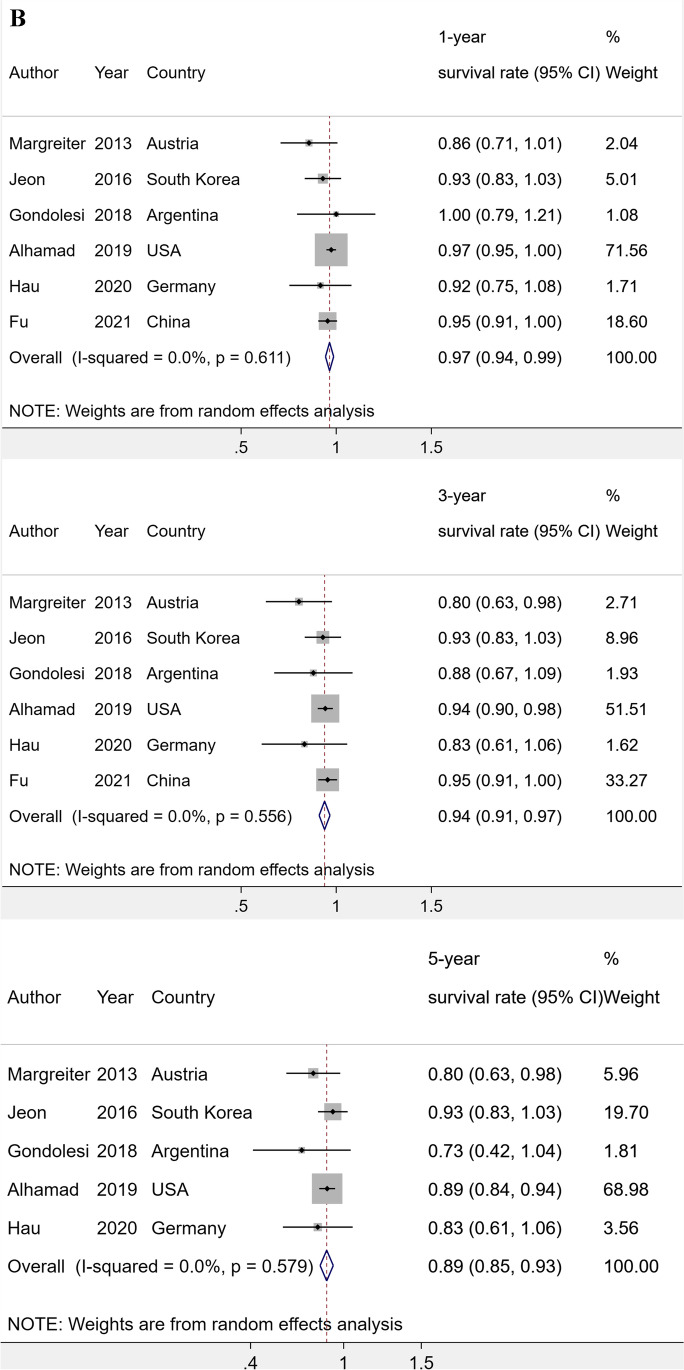

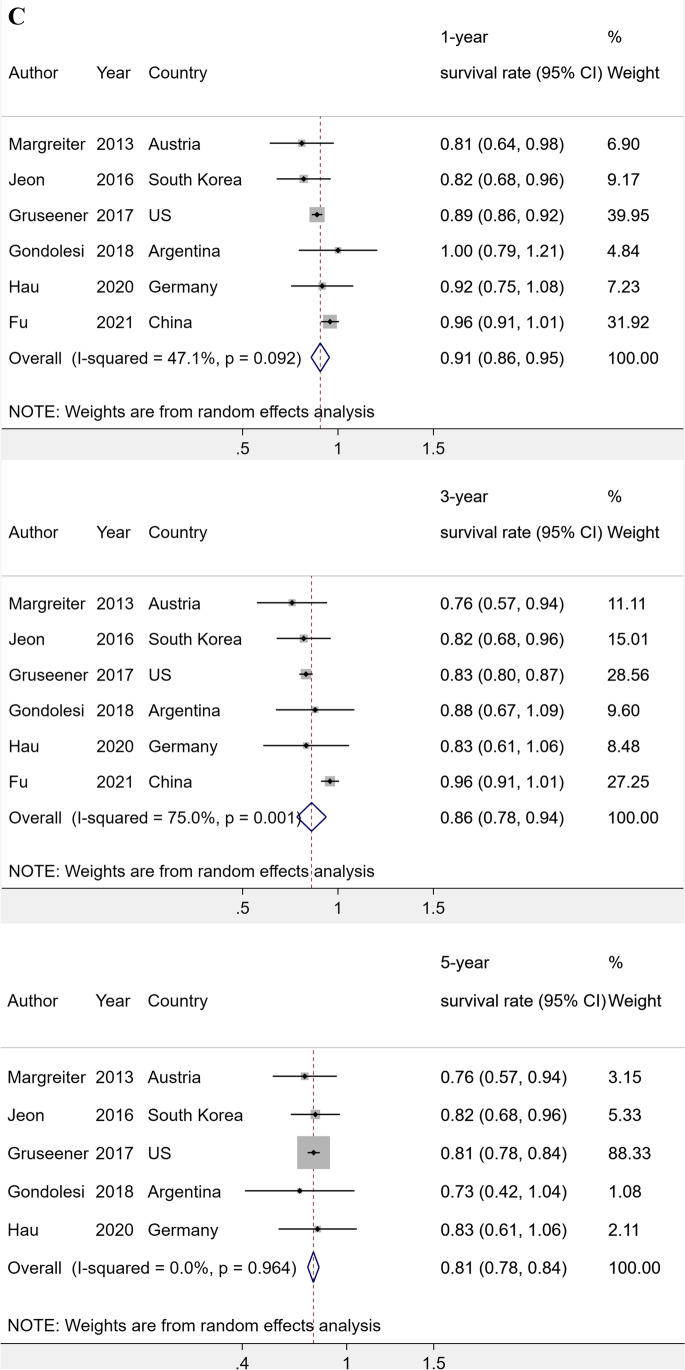
A Forest plot of meta-analysis of 1-year, 3-year, and 5-year patient survival rate after SPK. B Forest plot of meta-analysis of 1-year, 3-year, and 5-year kidney graft survival rates after SPK. C Forest plot of meta-analysis of 1-year, 3-year, and 5-year pancreas graft survival rates after SPK

Six studies [11, 16, 17, 19, 26–28] were included for meta-analysis of survival rates of SPK among T2DM recipients (Fig. 2A–C). The pooled 1-year, 3-year, and 5-year patient survival rates of T2DM combined with ESKD patients after SPK were 98% (95% confidence interval (CI), 96%–100%, I^2^ = 0%, p = 0.646), 95% (95%CI, 91%–99%, I^2^ = 39.6%, p = 0.142), and 91% (95%CI, 87%–96%, I^2^ = 0%, p = 0.438) (Fig. 2A). For kidney graft survival outcome, the synthesized 1-year, 3-year, and 5-year survival rates were 97% (95% CI, 94%–99%, I^2^ = 0%, p = 0.611), 94% (95% CI, 91%–97%, I^2^ = 0%, p = 0.556), and 89% (95%CI, 85%–93%, I^2^ = 0%, p = 0.579) (Fig. 2B). The heterogeneities among studies were slight.

The pooled 1-year, 3-year, and 5-year pancreas survival rates were 91% (95% CI, 86%–95%, I^2^ = 47.1%, p = 0.092), 86% (95% CI, 78%–94%, I^2^ = 75%, p = 0.001), and 81% (95%CI, 78%–84%, I^2^ = 0%, p = 0.964) (Fig. 2C). Since there was substantial heterogeneity in the 3-year survival rate analysis, sensitivity analyses were conducted and the result indicated that Fu et al.’ s study [27] was the reason of heterogeneity which might be due to the short median follow-up period (SDC-Figure S1).

#### Meta-analysis of patient and graft HR among T2DM compared with T1DM (Fig. 3A–C)

**Fig. 3:**
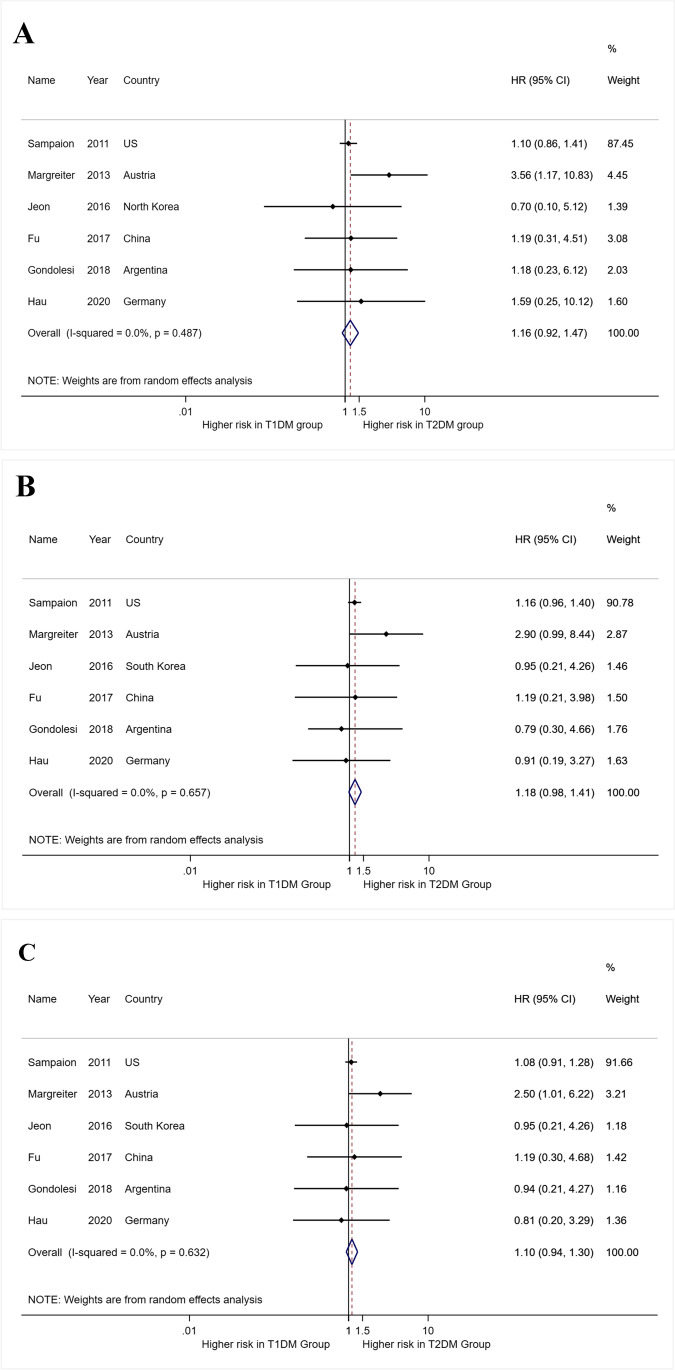
A Forest plot for meta-analysis of patient hazard ratios of T2DM compared with T1DM in SPK transplant recipients. B Forest plot for meta-analysis of kidney graft hazard ratios of T2DM compared with T1DM in SPK transplant recipients. C Forest plot for meta-analysis of pancreas hazard ratios of T2DM compared with T1DM in SPK transplant recipients

Six studies compared the survival rates between T1DM and T2DM [15–19, 28] (Fig. 3A–C). The pooled results indicated that T2DM has comparable survival estimates of patient death and graft failure with T1DM (for patient death, meta-hazard ratio (HR): 1.16, 95%CI, 0.92–1.47, I^2^ = 0%, p = 0.487; for kidney graft failure, meta-HR: 1.18, 95%CI, 0.98–1.41, I^2^ = 0%, p = 0.657; for pancreas graft failure, meta-HR: 1.10, 95%CI, 0.94–1.30, I^2^ = 0%, p = 0.632) (Fig. 3A–C).

#### Meta-analysis of patient and graft HR among T2DM after SPK compared with KTA (Fig. 4A–B)

**Fig. 4:**
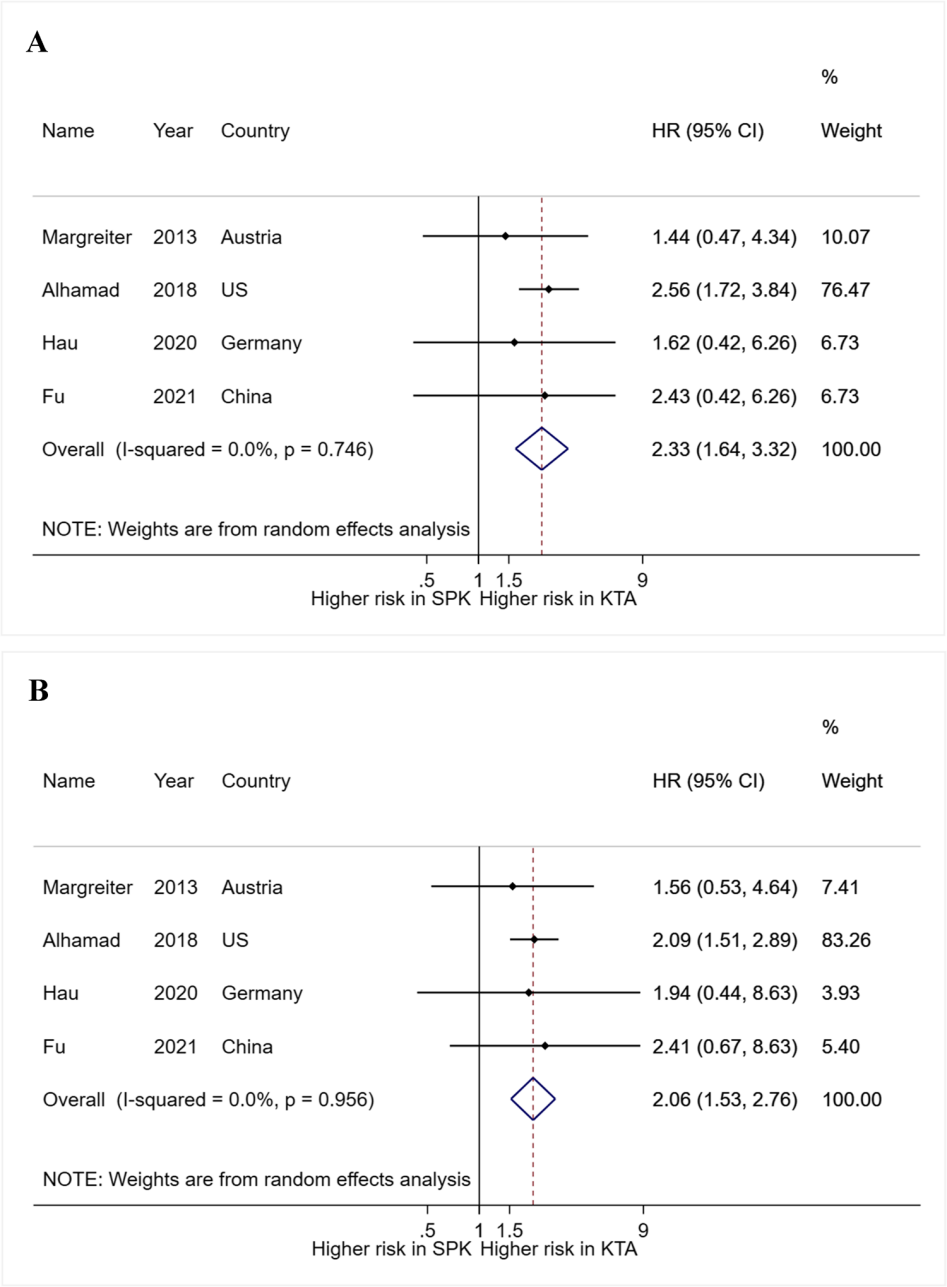
A Forest plot of meta-analysis of patient hazard ratios of SPK compared with KTA in T2DM transplant recipients. B Forest plot of meta-analysis of kidney hazard ratios of SPK compared with KTA in T2DM transplant recipients

Four studies were included for meta-analysis of survival outcome comparison between SPK and KTA among T2DM patients [16, 26–28] (Fig. 4A–B). The result indicated an increased survival risk after KTA among T2DM patients (pooled HR: 2.33, 95% CI, 1.64–3.32; I^2^ = 0%, p = 0.746) (Fig. 4A). In addition, renal survival outcome was superior in the SPK group as well (pooled HR, 2.06; 95% CI, 1.53–2.76; I^2^ = 0%, p = 0.956) (Fig. 4B).

#### Meta-analysis of complications RR among T2DM compared with T1DM (SDC-Figure S2)

The rates of rejection, infection, DGF of kidney graft and DGF of pancreas graft, and other types of complications were recorded and analyzed from included studies (Table 4). Four studies [15, 16, 18, 28] reported DGF data, 4 studies reported infection rates [15, 16, 18, 28], and 5 studies reported rejection data [15–18, 28] (Table 4 and SDC-Figure S2A-D). The risk of kidney graft DGF was significantly higher in the T2DM group (meta-RR: 1.47, 95%CI, 1.17–1.85, I^2^ = 0%, p = 0.935) compared with the T1DM group, while the risks of rejection, infection, pancreas graft, and DGF were comparable between the T2DM group and the T1DM group (SDC-Figure S2B-D). Since the heterogeneity of the analysis of rejection rates was above 50%, Hau et al.’s study [28] which reported the cumulative combined kidney and pancreas rejection rate might be the cause of diversity. A sensitivity analysis excluding Hau et al.’s study [28] was conducted; the result was presented in SDC-Figure S3.

**Table 4:** Main complications reported in included studies

First author and year	Rejection		DGF		Infection		Thrombosis	
	Definition	Rate (%)	Definition	Rate (%)	Definition	Rate (%)	Definition	Rate (%)
Sampaio[15], 2011	NR	SPK1: 15.8 SPK2: 14.7	NR	SPK1/SPK2: Kidney: 7.8/11.7	NR	SPK1/SPK2: 4.37/2.95	NR	NR
Margreiter[16], 2013	Clinical & biopsy	SPK1: 9.3 SPK2: 10 KTA2: 9.4	NR	SPK1/SPK2/KTA2: Pancreas: 16.4/15 Kidney: 12.3/15	NR	SPK1/SPK2/KTA2: 16.3/19/28.9	NR	NR
Jeon[17], 2016	NR	SPK1: 45 SPK2: 35.7	NR	NR	NR	NR	NR	NR
Fu[18], 2017	NR	SPK1: 13.9 SPK2: 5.5	NR	SPK1/SPK2: Pancreas: 0/13.7 Kidney: 2.8/5.5	NR	SPK1: 30.6 SPK2: 41.1	NR	NR
Gondolesi[19], 2018	Clinical diagnose	Kidney: 17.5	NR	Kidney: 21.7	NR	NR	NR	NR
Alhamad[26], 2019	NR	NR	NR	NR	NR	NR	NR	NR
Hau[28], 2020	**	SPK1/SPK2/KTA2: 21.3/58.3/15.44	Y	SPK1/SPK2/KTA2: Pancreas: 4.5/8.3/- Kidney: 14.9/16.7/42.3	NR	SPK1/SPK2: 6.7/8.3	NR	SPK1/SPK2: 10/9.1
Fu[27], 2021	Biopsy	SPK2/KTA2: 10.5/10.5	Y	SPK2/KTA2: 2.6/2.6	NR	SPK2/KTA2: 44.7/15.2	NR	NR

Notes: *E1 = era 1 from 1995–2001; E2 = era 2 from 2002–2008; E3 = era 3 from 2009–2015. **Combined cumulative kidney and pancreas rejection: increase of serum lipase and/or amylase, elevated fasting plasma glucose levels, a need for exogenous insulin, a low C-peptide level; an impaired renal function with elevated serum creatinine levels, clinical symptoms (pain, fever, leukocytosis); and/or confirmed by renal histology confirmed by renal histology. ***The need for insulin substitution at the time of hospital care but without further need after the discharge, the need for dialysis at hospital time but without further need after discharge

#### Meta-analysis of complications RR among SPK compared with KTA among T2DM patients (SDC-Figure S4)

Three studies reported cases of rejection, DGF of kidney graft, and infection in the SPK group and the KTA group among T2DM patients [16, 27, 28] (Table 4). The results indicated that the risks of kidney graft DGF and infection was not significantly higher in the KTA group (meta-RR of kidney graft DGF: 3.07, 95%CI, 1.37–6.89, I^2^ = 0%, p = 0.599; meta-RR of infection: 0.81, 95%CI, 0.33–2.01, I^2^ = 65.2%, p = 0.056) (SDC-Figure S4). A sensitivity analysis excluding Fu et al.’s [27] study was conducted. (SDC-Figure S4). The risk of developing rejection in the SPK group was not significantly higher (meta-RR: 0.55, 95%CI, 0.21–1.45, I^2^ = 38%, p = 0.199).

## Discussion

Previously, Chan et al. has attempted to address the question about the controversy of conducting SPK on T2DM patients in a review which vaguely concluded that the efficacy of SPK for T2DM remained controversial in 2016 [32]. Al-qaoud et al. concluded that the outcomes of strictly selected T2DM recipients mirrored those of T1DM in a literature review [33]. Hitherto, no high-quality evidence was available for T2DM patients with SPK, neither the precise survival risks of T2DM patients undertaking SPK compared with T1DM SPK patients or T2DM KTA patients. With several studies from different countries emerging between 2016 and 2020 [17, 18, 26–28], this study conducted a systematic review and meta-analysis to identify, collect, and synthesize all evidence reporting the survival outcomes of T2DM-ESKD recipients undertaking SPK worldwide, and make comparisons with their T1DM and KTA counterparts.

This systemic review included 9 studies comprising 811 T2DM-SPK recipients. The meta-5-year survival rates of patients and kidney grafts were above 90%, and the meta-5-year survival rate of pancreas graft was 81%. The survival outcomes of T2DM were identical to those of T1DM. For comparison of survival outcomes between SPK and KTA, the patients’ and grafts’ survival rates in the SPK group were superior to those in the KTA group. Although studies from a different geographical area with different organ distribution systems, the I^2^s were very low showing good homogeneity. The survival estimates of pancreas graft should be interpreted with caution given the various definitions reported in each program. Even though UNOS approved a standard definition in 2015 and the new policy was implemented in 2018, these were not reflected in the included studies [34–36].

The synthesized survival comparisons between T1DM and T2DM verified that the overall survival outcomes of T2DM recipients were comparable with those of T1DM, despite that the baseline characteristics of T1DM and T2DM were notably different, with T2MD recipients of older age and higher BMI [21, 29, 31, 32]. The explanation was complicated by the ambiguous classification of T1DM and T2DM. Concerning the specific selection criteria, the consensus remains lacking but continuous efforts were made. In this review, there were center-specific criteria in selecting T2DM SPK recipients. Their criteria were based on the guidelines of the American Diabetes Association (ADA) and the World Health Organization (WHO) which were useful but had limited effect on selecting or decision-making process to help transplant surgeons to identify which kind of patients would benefit from SPK [37]. Jeon et al.’s study added lower cardiovascular risk and BMI < 30 kg/m^2^ [17]. Gondolesi et al. added C-peptide > 2.35 ng/mL [17]. Hau et al. considered T2DM patients with age < 60 years, BMI < 30 kg/m^2^, and fasting C-p < 10 ng/mL [28]. Margreiter et al. [16] concluded that T2DM-ESKD patients with low coronary risk profile and age ≤ 55 years may have a favorable outcome from SPK. Previously, several attempts had been made for this issue. Sener et al. [38] had proposed criteria for pancreas transplantation in T2DM in 2010, suggesting C-peptide level, BMI, and pre-operation cardiovascular disease be considered. Dean et al. [38] mentioned T2DM with low insulin requirements would probably benefit from pancreas transplantation. Previously, mostly mentioned factors were C-peptide level, BMI, onset of DM [21–23], and recipient’s age [24]. While in 2018, UNOS amended the qualifying criteria and abandoned the maximal allowable BMI and C-peptide, and the policy was implemented in 2019 [33, 39]. Additionally, the novel subgroups of DM proposed in 2018 with a refined classification based on glutamate decarboxylase antibodies, age at diagnosis, BMI, HbA1c, and homeostatic model assessment 2 estimates of β-cell function and insulin resistance could also serve as a reference in making criteria [40].

For the comparison of SPK and KTA among T2DM patients, kidneys and patients’ survival outcomes after SPK were more favorable than those after KTA. The meta-HRs were dominated by Alhamad et al.’s study [26] with a weight of about 80%. Alhamad et al.’s study [26] was a retrospective design based on the national database, with multiple factors adjusted in the survival analysis. However, some covariables like the duration of diabetes, insulin dose before transplantation, waiting time, and other factors reflecting diabetes-related comorbidities of recipients and donor factors which might be significantly different were not reported and adjusted. Therefore, the significant hazard ratio of KTA compared with SPK should be interpreted with caution. Prospective randomized studies which could control for confounders were still lacking.

Surgical, infectious, and immunological complications after SPK have been tricky issues for a long period [41–45]. The present analysis indicated that the T2DM group had a higher risk of renal graft DGF. DGF of kidney graft was reported to be significantly associated with weight [15]. Most T2DM recipients were overweight or obese compared with T1DM patients, which could cause a higher DGF risk. The rejection rate, infection rate, and DGF rate of pancreas graft were not significantly inferior. The estimates were limited by the insufficient description of definitions of each complication and definition of pancreas graft failure, further hindering the comparison between groups. As pointed out by Dean et al. [6], currently, a lack of uniform definition regarding complications limited the broader application of collected data. The integrated results about complication risk ratio should be interpreted with caution.

## Strengths and limitations

This study has several strengths. The major one is that it is the first attempt to integrate the survival rate of patients and grafts of T2DM after SPK with software (Engauge Digitize) and HR calculation spreadsheet (by Tierney et al. [21]) with rigorous methodology. In addition, the studies included in this meta-analysis were drawn from a variety of countries that increased its applicability across many populations and organ transplantation centers. Next, there was slight between-study heterogeneity, indicating that estimates of mortality varied significantly beyond chance. There were some limitations, and the major one was that the absence of clear definitions of complications and pancreas graft failure hindered the interpretation of meta-results. Additionally, a small number of studies were enrolled in the meta-analysis of complications risk, and the meta-estimate should be interpreted with caution. Another main pitfall was that some included studies failed to provide multivariable-adjusted data, which might increase the risk of Type 2 error [46]. Besides, though capturing survival data from figures in articles made a quantitative synthesize of time-to-event data possible and had been used widely [47–49], still it would have a slight degree of error. Therefore, transparency of original studies is advocated.

## Conclusions

The synthesized survival estimates of T2DM-ESKD patients after SPK were above 90%. Specifically, survival outcomes of T2DM patients are comparable with that of T1DM, and for T2DM, SPK is superior to KTA. However, a uniform criterion of T2DM subsets that would benefit the most from SPK and clearly defined diagnosis standards of SPK-related complications are urgent to be made.

## Availability of data and code

The datasets or code used or analyzed during the current study are available from the corresponding author on reasonable request.

## Supplementary Information

Below is the link to the electronic supplementary material.

Supplementary file1 (DOCX 4071 KB)

## Abbreviations

SPK: Simultaneous pancreas-kidney transplantation
ESKD: End-stage kidney disease
DM: Diabetes mellitus
T2DM: Type 2 diabetes mellitus
T1DM: Type 1 diabetes mellitus
KTA: Kidney transplantation alone
CI: Confidence interval
CNKI: China National Knowledge Infrastructure
