# Supplementary material for: Simultaneous pancreas and kidney transplantation for end-stage kidney disease patients with type 2 diabetes mellitus: a systematic review and meta-analysis
Source: Langenbecks Arch Surg. 2021 Jul 19;407(3):909–25. doi: 10.1007/s00423-021-02249-y (PMC9151548; doi:10.1007/s00423-021-02249-y)
Supplement: Supplementary file 1 — Supplementary file1 (DOCX 4071 KB) [file 423_2021_2249_MOESM1_ESM.docx]

**Supplemental digital content**

**Supplemental Table Legend**

Table S1. Search strategy for each database

Table S2: Excluded 8 studies due to overlapping samples

Table S3: Additional information (pre-comorbidities, induction and maintenance regimen) of included 8 studies.

Table S4. MODIFIED NEWCASTLE - OTTAWA QUALITY ASSESSMENT SCALE—Cohort Study

**Supplemental Figure Legend**

Figure S1. Sensitivity analysis by excluding Fu et al.’s study in meta-analysis of 3-year pancreas survival rate after SPK

Figure S2: Forest plot for meta-analysis of risk ratio of complications betweenT1DM and T2DM. A, DGF of kidney graft; B, DGF of pancreas graft; C, Infection; D, Rejection.

Figure S3. Sensitivity analysis by excluding Hau et al.’s study in meta-analysis of rejection risk of T2DM compared with T1DM

Figure S4. Forest plot for meta-analysis of risk ratio of complications between KTA and SPK

Table S1. Search strategy for each database

| Search strategy for PubMed | |
| --- | --- |
| Search terms | |
| #1 | (pancreas transplantation [MeSH Terms]) OR (((((((Grafting, Pancreas) OR Graftings, Pancreas) OR Pancreas Grafting) OR Pancreas Graftings) OR Transplantation, Pancreas) OR Pancreas Transplantations) OR Transplantations, Pancreas) |
| #2 | ("Kidney Transplantation"[Mesh]) OR (((((((((Renal Transplantation) OR Renal Transplantations) OR Transplantations, Renal) OR Transplantation, Renal) OR Grafting, Kidney) OR Kidney Grafting) OR Transplantation, Kidney) OR Kidney Transplantations) OR Transplantations, Kidney) |
| #3 | (((((((((((((Simultaneous Pancreas-kidney transplantation? OR "Simultaneous kidney-Pancreas transplantation") OR "Renal-pancreas transplant") OR "Simultaneous renal transplantation") OR ("Simultaneous transplantation of kidney and pancreas")) OR "Simultaneous pancreas kidney transplant") OR "Kidney-pancreas transplants") OR ("Combined renal and pancreatic transplantation")) OR "combined pancreas-kidney transplantation") OR SPK) OR "pancreas kidney transplantation") OR "pancreas kidney transplantations") OR "kidney pancreas transplantation") OR "kidney pancreas transplantationS" |
| #4 | ("Diabetes Mellitus, Type 2"[Mesh]) OR ((((((((((((((((((((((((((((((((Diabetes Mellitus, Noninsulin-Dependent) OR Diabetes Mellitus, Ketosis-Resistant) OR Diabetes Mellitus, Ketosis Resistant) OR Ketosis-Resistant Diabetes Mellitus) OR Diabetes Mellitus, Non Insulin Dependent) OR Diabetes Mellitus, Non-Insulin-Dependent) OR Non-Insulin-Dependent Diabetes Mellitus) OR Diabetes Mellitus, Stable) OR Stable Diabetes Mellitus) OR Diabetes Mellitus, Type II) OR Diabetes Mellitus, Noninsulin Dependent) OR NIDDM) OR Diabetes Mellitus, Maturity-Onset) OR Maturity-Onset Diabetes Mellitus) OR Diabetes Mellitus, Maturity Onset) OR Maturity Onset Diabetes Mellitus) OR MODY) OR Diabetes Mellitus, Slow-Onset) OR Diabetes Mellitus, Slow Onset) OR Slow-Onset Diabetes Mellitus) OR Type 2 Diabetes Mellitus) OR Noninsulin-Dependent Diabetes Mellitus) OR Noninsulin Dependent Diabetes Mellitus) OR Maturity-Onset Diabetes) OR Diabetes, Maturity-Onset) OR Maturity Onset Diabetes) OR Type 2 Diabetes) OR Diabetes, Type 2) OR Diabetes Mellitus, Adult-Onset) OR Adult-Onset Diabetes Mellitus) OR Diabetes Mellitus, Adult Onset) OR Diabetes Mellitus, Type 2) Sort by: [pubsolr12] |
| #5 | (#1 & #2) or #3 |
| #6 | #4 and #5 |

| Search strategy for Cochrane | |
| --- | --- |
| Search terms | |
| #1 | MeSH descriptor: [Kidney Transplantation] explode all trees |
| #2 | MeSH descriptor: [Pancreas Transplantation] explode all trees |
| #3 | (Renal Transplantation) (Word variations have been searched) |
| #4 | (renal) (Word variations have been searched) |
| #5 | (kidney) (Word variations have been searched) |
| #6 | (kidneys) (Word variations have been searched) |
| #7 | (transplantation) (Word variations have been searched) |
| #8 | (transplantations) (Word variations have been searched) |
| #9 | (transplant) (Word variations have been searched) |
| #10 | (graft) (Word variations have been searched) |
| #11 | (grafting) (Word variations have been searched) |
| #12 | (graftings) (Word variations have been searched) |
| #13 | (graftings) (Word variations have been searched) |
| #14 | (diabet*) (Word variations have been searched) |
| #15 | MeSH descriptor: [Diabetes Mellitus, Type 2] explode all trees |
| #16 | (NIDDM OR "II DM" OR DM2) (Word variations have been searched) |
| #17 | #4 OR #5 OR #6 |
| #18 | #7 OR #8 OR #9 OR #10 OR #11 OR #12 OR #13 |
| #19 | #14 OR #15 OR #16 |
| #20 | (PANCREA*) (Word variations have been searched) |
| #21 | (PANCREAS) (Word variations have been searched) |
| #22 | #20 OR #21 |
| #23 | #22 AND #18 |
| #24 | #23 OR #2 |
| #25 | #17 AND #18 |
| #26 | #25 OR #1 |
| #27 | #24 AND #26 AND #19 |

| Search strategy for Embase | |
| --- | --- |
| Search terms | |
| #1 | 'kidney transplantation'/exp |
| #2 | renal transplantations' OR 'renal transplantation' OR 'transplantations, renal' OR 'transplantation, renal' OR 'grafting, kidney' OR 'kidney grafting' OR 'transplantation, kidney' OR 'kidney transplantations' OR 'transplantations, kidney' |
| #3 | kidney pancreas transplantation'/exp |
| #4 | simultaneous pancreas-kidney transplantation' OR 'simultaneous kidney-pancreas transplantation' OR 'renal-pancreas transplant' OR 'simultaneous renal transplantation' OR 'simultaneous transplantation of kidney and pancreas' OR 'simultaneous pancreas–kidney transplant' OR 'kidney-pancreas transplants' OR 'combined renal and pancreatic transplantation' OR 'combined pancreas-kidney transplantation' OR 'spk' OR 'pancreas kidney transplantation' OR 'pancreas kidney transplantations' OR 'kidney pancreas transplantation' OR 'kidney pancreas transplantations' |
| #5 | 'pancreas transplantation'/exp |
| #6 | 'grafting, pancreas' OR 'graftings, pancreas' OR 'pancreas grafting' OR 'pancreas graftings' OR 'transplantation, pancreas' OR 'pancreas transplantations' OR 'transplantations, pancreas' |
| #7 | #5 OR #6 |
| #8 | #3 OR #4 |
| #9 | #1 OR #2 |
| #10 | 'diabetes mellitus, noninsulin-dependent' OR 'diabetes mellitus, ketosis-resistant' OR 'diabetes mellitus, ketosis resistant' OR 'ketosis-resistant diabetes mellitus' OR 'diabetes mellitus, non insulin dependent' OR 'diabetes mellitus, non-insulin-dependent' OR 'non-insulin-dependent diabetes mellitus' OR 'diabetes mellitus, stable' OR 'stable diabetes mellitus' OR 'diabetes mellitus, type ii' OR niddm OR 'diabetes mellitus, noninsulin dependent' OR 'diabetes mellitus, maturity-onset' OR 'diabetes mellitus, maturity onset' OR 'maturity-onset diabetes mellitus' OR 'maturity onset diabetes mellitus' OR mody OR 'diabetes mellitus, slow-onset' OR 'diabetes mellitus, slow onset' OR 'slow-onset diabetes mellitus' OR 'type 2 diabetes mellitus' OR 'noninsulin-dependent diabetes mellitus' OR 'noninsulin dependent diabetes mellitus' OR 'maturity-onset diabetes' OR 'diabetes, maturity-onset' OR 'maturity onset diabetes' OR 'type 2 diabetes' OR 'diabetes, type 2' OR 'diabetes mellitus, adult-onset' OR 'adult-onset diabetes mellitus' OR 'diabetes mellitus, adult onset' |
| #11 | non insulin dependent diabetes mellitus'/exp |
| #12 | #10OR #11 |
| #13 | #7 AND #9 |
| #14 | #8 OR #13 |
| #15 | #12 AND #14 |

| Search strategy for OCLC | |
| --- | --- |
| Search terms | |
| #1 | (mh= "pancreas transplantation") or  (kw: grafting and kw: pancreas) or ((kw: graftings and kw: pancreas)) or ((kw: transplantation and kw: pancreas)) or ((kw: transplantations and kw: pancreas)) |
| #2 | (mh= "kidney transplantation") or (kw: renal and kw: transplantations) or ((kw: renal and kw: transplantation)) or ((kw: grafting and kw: kidney)) or ((kw: transplantation and kw: kidney)) or ((kw: transplantations and kw: kidney)) |
| #3 | (mh= "kidney pancreas transplantation") or ((su="simultaneous pancreas-kidney transplantation") or (su="simultaneous kidney-pancreas transplantation") or (su= "renal-pancreas transplant") or (su= "simultaneous renal transplantation") or (su= "simultaneous transplantation of kidney and pancreas") or (su= "simultaneous pancreas–kidney transplant") or (su= "kidney-pancreas transplants") or (su= "combined renal and pancreatic transplantation") or (su= "combined pancreas-kidney transplantation") or su= "spk" or (su= "pancreas kidney transplantation") or (su= "pancreas kidney transplantations")) |
| #4 | (mh= "non insulin dependent diabetes mellitus") or ((su= "Diabetes Mellitus" and su= "Noninsulin-Dependent") or (su= "Diabetes Mellitus" and su= "Ketosis-Resistant") or (su= "Diabetes Mellitus" and su= "Ketosis Resistant") or (su= "Ketosis-Resistant Diabetes Mellitus") or (su= "Diabetes Mellitus" and su= "Non Insulin Dependent") or (su= "Diabetes Mellitus" and su= "Non-Insulin-Dependent") or (su= "Non-Insulin-Dependent Diabetes Mellitus") or (su= "Diabetes Mellitus" and su= "Stable") or (su= "Stable Diabetes Mellitus") or (su= "Diabetes Mellitus" and su= "Type II") or su= "NIDDM" or (su= "Diabetes Mellitus" and su= "Noninsulin Dependent") or (su= "Diabetes Mellitus" and su= "Maturity-Onset") or (su= "Diabetes Mellitus" and su= "Maturity Onset") or (su= "Maturity-Onset Diabetes Mellitus") or (su= "Maturity Onset Diabetes Mellitus") or su= "MODY" or (su= "Diabetes Mellitus" and su= "Slow-Onset") or (su= "Diabetes Mellitus" and su= "Slow Onset") or (su= "Slow-Onset Diabetes Mellitus") or (su= "Diabetes Mellitus" and su= "Maturity Onset") or (su= "”type 2 Diabetes Mellitus”") OR (su= "” Noninsulin-Dependent Diabetes Mellitus”") or (su= "” Noninsulin Dependent Diabetes Mellitus”") or (su= "” Maturity-Onset Diabetes”") or (su= "“Maturity Onset Diabetes”") or (su= "Diabetes" and su= "Maturity-Onset") or (su= "Diabetes" and su= "type 2") or (su= "Diabetes Mellitus" and su= "Adult-Onset") and (su= "Diabetes Mellitus" and su= "Adult Onset") or (su= "” Adult-Onset Diabetes Mellitus”") or (su= "” Type 2 Diabetes”")) |
| #5 | (#1 & #2) or #3 |
| #6 | #4 and #5 |

| Search strategy for EBSCO | |
| --- | --- |
| Search terms | |
| #1 | 'pancreas transplantation' OR 'grafting, pancreas' OR 'graftings, pancreas' OR 'pancreas grafting' OR 'pancreas graftings' OR 'transplantation, pancreas' OR 'pancreas transplantations' OR 'transplantations, pancreas' |
| #2 | 'kidney transplantation ' OR 'renal transplantations' OR 'renal transplantation' OR 'transplantations, renal' OR 'transplantation, renal' OR 'grafting, kidney' OR 'kidney grafting' OR 'transplantation, kidney' OR 'kidney transplantations' OR 'transplantations, kidney' |
| #3 | 'simultaneous pancreas-kidney transplantation' OR 'simultaneous kidney-pancreas transplantation' OR 'renal-pancreas transplant' OR 'simultaneous renal transplantation' OR 'simultaneous transplantation of kidney and pancreas' OR 'simultaneous pancreas–kidney transplant' OR 'kidney-pancreas transplants' OR 'combined renal and pancreatic transplantation' OR 'combined pancreas-kidney transplantation' OR 'spk' OR 'pancreas kidney transplantation' OR 'pancreas kidney transplantations' OR 'kidney pancreas transplantation' OR 'kidney pancreas transplantations' |
| #4 | ‘Diabetes Mellitus, Type 2' OR ‘Diabetes Mellitus, Noninsulin-Dependent’ OR ‘Diabetes Mellitus, Ketosis-Resistant’ OR ‘Diabetes Mellitus, Ketosis Resistant’ OR ‘Ketosis-Resistant Diabetes Mellitus’ OR ‘Diabetes Mellitus, Non Insulin Dependent’ OR ‘Diabetes Mellitus, Non-Insulin-Dependent’ OR ‘Non-Insulin-Dependent Diabetes Mellitus’ OR ‘Diabetes Mellitus, Stable’ OR ‘Stable Diabetes Mellitus’ OR ‘Diabetes Mellitus, Type II’ OR ‘NIDDM’ OR ‘Diabetes Mellitus, Noninsulin Dependent’ OR ‘Diabetes Mellitus, Maturity-Onset’ OR ‘Diabetes Mellitus, Maturity Onset’ OR ‘Maturity-Onset Diabetes Mellitus’ OR ‘Maturity Onset Diabetes Mellitus’ OR ‘MODY’ OR ‘Diabetes Mellitus, Slow-Onset’ OR ‘Diabetes Mellitus, Slow Onset’ OR ‘Slow-Onset Diabetes Mellitus’ OR ‘Type 2 Diabetes Mellitus’ OR ‘Noninsulin-Dependent Diabetes Mellitus’ OR ‘Noninsulin Dependent Diabetes Mellitus’ OR ‘Maturity-Onset Diabetes’ OR ‘Diabetes, Maturity-Onset’ OR ‘Maturity Onset Diabetes’ OR ‘Type 2 Diabetes’ OR ‘Diabetes, Type 2’ OR ‘Diabetes Mellitus, Adult-Onset’ OR ‘Adult-Onset Diabetes Mellitus’ OR ‘Diabetes Mellitus, Adult Onset’ |
| #5 | (#1 & #2) or #3 |
| #6 | #4 and #5 |

| Search strategy for web of science | |
| --- | --- |
| Search terms | |
| #1 | (TS=pancreas transplantation) OR ( TS= grafting, pancreas ) OR ( TS= graftings, pancreas ) OR ( TS= pancreas grafting ) OR ( TS= pancreas graftings ) OR ( TS= transplantation, pancreas ) OR ( TS= pancreas transplantations ) OR ( TS= transplantations, pancreas ) |
| #2 | (TS=kidney transplantation) OR ( TS= renal transplantations ) OR ( TS= renal transplantation ) OR ( TS= transplantations, renal ) OR ( TS= transplantation, renal ) OR ( TS= grafting, kidney ) OR ( TS= kidney grafting ) OR ( TS= transplantation, kidney ) OR ( TS= kidney transplantations ) OR ( TS= transplantations, kidney ) |
| #3 | TOPIC: (simultaneous pancreas-kidney transplantation) OR TOPIC:  (simultaneous kidney-pancreas transplantation) OR TOPIC: (simultaneous renal transplantation) OR TOPIC: (simultaneous transplantation of kidney and pancreas) OR TOPIC: (simultaneous pancreas kidney transplant) OR  TOPIC: (kidney-pancreas transplants) OR TOPIC: (combined renal and pancreatic transplantation) OR TOPIC: (combined pancreas-kidney transplantation) OR TOPIC: (pancreas kidney transplantation) OR TOPIC:  (pancreas kidney transplantations) OR TOPIC: (kidney pancreas transplantation) OR TOPIC: (kidney pancreas transplantations) |
| #4 | (TS = Diabetes Mellitus,Type 2) OR ( TS= Diabetes Mellitus, Noninsulin-Dependent ) OR ( TS= Diabetes Mellitus, Ketosis-Resistant ) OR ( TS= Diabetes Mellitus, Ketosis Resistant ) OR ( TS= Ketosis-Resistant Diabetes Mellitus ) OR ( TS= Diabetes Mellitus, Non Insulin Dependent ) OR ( TS= Diabetes Mellitus, Non-Insulin-Dependent ) OR ( TS= Non-Insulin-Dependent Diabetes Mellitus ) OR ( TS= Diabetes Mellitus, Stable ) OR ( TS= Stable Diabetes Mellitus ) OR ( TS= Diabetes Mellitus, Type II ) OR ( TS= NIDDM ) OR ( TS= Diabetes Mellitus, Noninsulin Dependent ) OR ( TS= Diabetes Mellitus, Maturity-Onset ) OR ( TS= Diabetes Mellitus, Maturity Onset ) OR ( TS= Maturity-Onset Diabetes Mellitus ) OR ( TS= Maturity Onset Diabetes Mellitus ) OR ( TS= MODY ) OR ( TS= Diabetes Mellitus, Slow-Onset ) OR ( TS= Diabetes Mellitus, Slow Onset ) OR ( TS= Slow-Onset Diabetes Mellitus ) OR ( TS= Type 2 Diabetes Mellitus ) OR ( TS= Noninsulin-Dependent Diabetes Mellitus ) OR ( TS= Noninsulin Dependent Diabetes Mellitus ) OR ( TS= Maturity-Onset Diabetes ) OR ( TS= Diabetes, Maturity-Onset ) OR ( TS= Maturity Onset Diabetes ) OR ( TS= Type 2 Diabetes ) OR ( TS= Diabetes, Type 2 ) OR ( TS= Diabetes Mellitus, Adult-Onset ) OR ( TS= Adult-Onset Diabetes Mellitus ) OR ( TS= Diabetes Mellitus, Adult Onset ) |
| #5 | (#1 & #2) or #3 |
| #6 | #4 and #5 |

| Search strategy for CNKI(Chinese database) | |
| --- | --- |
| Search terms | |
| #1 | SU=胰腺移植 OR SU=胰移植 OR SU=移植，胰 OR SU=移植，胰腺 |
| #2 | SU=肾移植 OR SU=肾脏移植 OR SU=移植，肾脏 OR SU=移植，肾 |
| #3 | SU=胰肾联合移植 OR SU=肾胰联合移植 OR SU=肾脏胰脏联合移植 |
| #4 | SU='糖尿病,2型' OR SU='糖尿病,二型' OR SU='糖尿病,II型' OR SU='二型糖尿病' OR SU='2型糖尿病' OR SU='II型糖尿病' |
| #5 | (#1 & #2) or #3 |
| #6 | #4 and #5 |

| Search strategy for CNKI(Translated version) | |
| --- | --- |
| Search terms | |
| #1 | SU=pancreas transplant OR SU=pancreas transplantation OR SU=transplant, pancreas OR SU=transplantation, pancreas |
| #2 | SU=kidney transplant OR SU=kidney transplantation OR SU=transplant, kidney OR SU=transplantation,kidney |
| #3 | SU=simultaneous pancreas kidney transplantation OR SU= simultaneous kidney pancreas transplantation OR SU=simultaneous kidney-pancreas transplantation |
| #4 | SU='diabetes, type 2' OR SU='diabetes, 2' OR SU='diabetes, type II' OR SU='type II diabetes' OR SU='type 2 diabetes' OR SU='class II diabetes' |
| #5 | (#1 & #2) or #3 |
| #6 | #4 and #5 |

| Search strategy for CBM | |
| --- | --- |
| Search terms | |
| #1 | "肾移植"[扩展:不加权] |
| #2 | "胰腺移植"[扩展:不加权] |
| #3 | "胰肾联合移植"[全字段:智能] |
| #4 | "糖尿病, 2型"[不加权:扩展] |
| #5 | ((("移植术,肾"[全字段:智能]) OR "肾移植术"[全字段:智能]) OR "移植,肾"[全字段:智能]) OR "移植,肾脏"[全字段:智能] |
| #6 | ("移植术,胰腺"[全字段:智能]) OR "移植,胰腺"[全字段:智能] |
| #7 | (((((((((("糖尿病,成年型"[全字段:智能]) OR "糖尿病,非酮症性"[全字段:智能]) OR "糖尿病,成熟型"[全字段:智能]) OR "糖尿病,非胰岛素依赖型"[全字段:智能]) OR "糖尿病,慢发作型"[全字段:智能]) OR "糖尿病,稳定性"[全字段:智能])) OR "糖尿病,Ⅱ型"[全字段:智能]) OR "成熟型糖尿病"[全字段:智能]) OR "MODY"[全字段:智能]) OR "NIDDM"[全字段:智能] |
| #8 | (#1) OR (#5) |
| #9 | (#2) OR (#6) |
| #10 | (#8) AND (#9) |
| #11 | (#10)OR (#3) |
| #12 | (#7)OR (#4) |
| #13 | (#12) AND (#11) |

| Search strategy for CBM(Translated version) | |
| --- | --- |
| Search terms | |
| #1 | "kidney transplantation"[Exp:Wtd] |
| #2 | "pancreas transplantation"[Exp:Wtd] |
| #3 | "simultaneous pancreas kidney transplantation"[full fields] |
| #4 | "diabetes, type 2"[unWtd:Exp] |
| #5 | ((("transplantation, kidney"[full fields]) OR "kidney transplant"[full fields]) OR "transplant, kidney"[full fields]) OR " kidney transplantation"[full fields] |
| #6 | ("transplantation, pancreas"[full fields]) OR "grafting, pancreas"[full fields] |
| #7 | (((((((((("Diabetes Mellitus, Adjult-Onset "[full fields]) OR " Diabetes Mellitus, Ketosis-Resistant "[full fields]) OR " Diabetes Mellitus, Maturity-Onset "[full fields]) OR " Diabetes Mellitus, Noninsulin Dependent "[full fields]) OR " Diabetes Mellitus, Slow Onset "[full fields]) OR " Diabetes Mellitus, Stable "[full fields])) OR "diabetes, type 2"[full fields]) OR " Maturity-Onset Diabetes "[full fields]) OR "MODY"[full fields]) OR "NIDDM"[full fields] |
| #8 | (#1) OR (#5) |
| #9 | (#2) OR (#6) |
| #10 | (#8) AND (#9) |
| #11 | (#10)OR (#3) |
| #12 | (#7)OR (#4) |
| #13 | (#12) AND (#11) |

**Table S2. MODIFIED NEWCASTLE - OTTAWA QUALITY ASSESSMENT SCALE**

**COHORT STUDIES**

**Selection**

1) Representativeness of the exposed cohort (Max:**🟑🟑**)

a) sample from single centre and sample size ^#^ group <50

b) sample from more than one centre or sample size belong to a range of 50-100**🟑**

c) sample from the national database or sample size >100**🟑🟑**

2) Selection of the non-exposed cohort(Max:**🟑**)

a) drawn from the same hospital/database as the exposed cohort **🟑**

b) drawn from a different source

c) no description of the derivation of the non-exposed cohort

3) Ascertainment of exposure(Max:**🟑**)

a) secure record (eg surgical records) **🟑**

b) written self-report

c) no description

4) Study was published within 5 years (after 2016) (Max:**🟑**)

a) yes **🟑**

b) no

**Comparability**

1) Comparability of cohorts on the basis of the design or analysis (Max:**🟑**)

a) study providing adjusted HR (T1DM vs. T2DM or SPK vs. KTA) **🟑**

b) study with unadjusted HR

**Outcome**

1) Assessment of outcome (Max:**🟑**)

a) record linkage **🟑**

b) self report

c) no description

2) Was follow-up long enough for outcomes to occur (Max:**🟑🟑**)

a) with mean/median follow up period > 3 years or longest follow up period >10 years**🟑**

or b) for studies with mean/median follow up period > 5 years or longest follow up period >15 years**🟑🟑**

3) Adequacy of follow up of cohorts (Max:**🟑🟑**)

a) complete follow up - all subjects accounted for **🟑**

b) subjects lost to follow up unlikely to introduce bias - small number lost - > _70_ % (select an adequate %) follow up, or description provided of those lost) **🟑**

c) follow up rate < ____% (select an adequate %) and no description of those lost

d) no statement

Notes： # the sample size only for T2DM patients undertaking SPK.

Table S3: 8 Excluded studies with overlapping samples

| **First author,**  **Year, Country** | **Study design** | **Data sources** | **No. cases** | **Study period** | **Mean/median age, y** | **BMI** | **sex (M%)** | **Duration of DM(y)** | **follow-up** |  |
| --- | --- | --- | --- | --- | --- | --- | --- | --- | --- | --- |
| Chakkera, 2010,  USA | Cohort  study | Mayo Clinic Hospital | SPT1:70 SPT2:10 | 2003/10-2008/09 | SPT1: 44±11 SPT2: 51±9* | SPT1: 24.8±4.2 SPT2: 27±3* | SPT1:88.6* SPT2:90 | SPT1: 29±9 SPT2: 19±10** | M:485d |  |
| Gruessner, 2017,  USA | Cohort  study | IPTR/UNOS | SPT2:1322 | 1995-2015 | SPT2:46.4±8.3 | <18.5: 24 18.5–24.9: 535 25–29.9:540 ≥30:44 | 70.80 | SPT2: 21.1±7.9 | Up to 20y |  |
| Light,  2013,  USA | Cohort  study | Washington Hospital Center | SPT1:115 SPT2:58 KTAT2:24 | 1989-2008 | SPT1:38.5±7.96** SPT2:42.8±8.4 KTAT2:58.2±7.01@@ | SPT1:23.1±3.4** SPT2:26.09±4 KTAT2:NA | T1DM:65.2 T2DM:65.5* KTA:83.3@ | SPT1: 22.57±7.16** SPT2: 19.19±7.08 | Median: 4.08y | |
| Wiseman^a^, 2012,  USA | Cohort  study | IPTR/UNOS | SPT2:424 KTAT2:4005 LDT2:1987 | 2000-2008 | KTA/SPK/LDKA**@@ 18–34y:1.6%/6.1%/2.9% 35-49y:26.1%54.0%30.4% 50-59y:72.3%39.9%66.7% | SPT2:24.7±2.8**@@ KTAT2:25.8±2.7 LDT2:25.9±2.7 | SPT2:68.9*@ KTAT2:68 LDT2:68.1 | NR | Up to 8y | |
| Singh, 2008  USA | Cohort  study | Wake Forest University Baptist Medical Center | Cp>2:7 Cp<2:67 | 2002/1-2007/1 | Cp <2 : 51±2.9** Cp >2 : 41±1.1 | NR | Cp>2:42.9* Cp<2:41.8 | Cp >2: 23±0.94* Cp <2:15±1.7 | Cp>2:40m* Cp<2:40m | |
| Gruessner,  2018,  USA | Cohort  study | IPTR/UNOS | SPT1: 11407 SPT2:1136 | 2001-2016 | <18: 0.03%  18–29: 7.41%  30–44: 57.07%  45–59: 34.31%  >60: 1.16% | <18.5: 1.99%  18.5–24.9: 50.39%  25–29.9: 35.39%  >30: 11.73% | SPK: 62.38 * | NR | at least 6m | |
| Andacoglu,  2019,  USA | cohort study | MedStar Georgetown Transplant Institute | SPT1:27  SPT2:9 | 2013-2016 | SPT1: 36.9±7.8* SPT2: 53.7±7.3 | NR | NR | NR | 2Y | |
| Pham, 2020,  USA | Cohort study | Wisconsin Hospital | SPK1:284  SPK2:39 | 2006-2017 | SPK1: 42.4 ± 9.0* SPK2: 47.0 ± 9.1 | SPK1:59.9* SPK2:85 | SPK1: 25.6 ± 3.7* SPK2: 27.0 ± 3.4 | SPK1: 28.8 SPK2:19.3* | Mean:  5y | |

Note: SPT1=T1DM recipients with SPK; SPT2= T2DM recipients with SPK; KTAT1= T1DM recipients undertaking deceased kidney transplantation alone; Cp= C-peptipe level (ng/ml); NR= not reported; CHD= coronary heart disease; AOD= Arterial obstructive disease; HBP= high blood pressure; CsA= cyclosporine A; AND= autonomic dysfunction; ATG= rabbit anti-thymocyte; ALG= anti-lymphocyte globulin; OKT3= muronab-anti CD3 antibody; ATG= rabbit anti-thymocyte globulin; IL2-RA= interleukin 2 receptor antibody (basiliximab and daclizumab); AP= antiproliferative; MMF= mycophenolate mofetil (includes nonenteric and enteric formulations); SRL= sirolimus; AZA= azathioprine; CAD= coronary artery disease; CVD= cerebrovascular disease; PVD= peripheral vascular disease; globulin; RTP=retinopathy; NRP= Neuropahty; ^#1^= withdrawn by postoperative day 4; ^#2^=32 underwent early steroid elimination; *=there was no statistical significant between SPT1 and SPT2, **=there was statistical significant between SPT1 and SPT2, @=there was no statistical significant between SPT2 and KTAT2, @@=there was statistical significant between SPT1 and KTAT2. ***P < 0.05 for chi‐squared tests comparing differences between SPK,P+, SPK,P‐, DD‐KA, and LD‐KA groups. ^ RTP:85.7%/50%** NRP:60%/80%* AND: 50%/50%*

Table S4: Additional information of included 8 studies.

| **First author,**  **Year,**  **Country** | **Pre-operation comorbidities (%)** | **Induction (%)** | | **Maintenance regimen (%)** |
| --- | --- | --- | --- | --- |
| Sampaio, 2011, USA | SPK1/SPK2  HBP: 79.1/71.9** CAD:10.2/10.5* CVD: 2.2/2.2* PVD: 7.0/6.2* | SPK1/SPK2** ALG/OKT3:3.3/2.4 ATG:37.3/40.9 IL2-RA:24.2/10.8  Others:38.5/46.3 | SPK1/SPK2** CsA:8.9/4.5 Tac:84.7/86.6; MMF:75.9/ 76.6 SRL:11.3/7.0 | |
| Margreiter, 2013, Austria | SPKT1/SPKT2/KTA2(*@@):  AOD:23.6/76.2/59.4 RTP: 13.4/76.1/50 NRP:7.7/71.4/15.6 CHD:30.3/57.1/81.2 | ATG | Tac, MMF; rapid steroid-tapering regimen; | |
| Jeon, 2016, South Korea | SPK/LDKT/KTA  CHD: 17.7/16/17.6* | IL2-RA | Corticosteroid, CNI, MMF | |
| Fu, 2017, China | NR | NR | NR | |
| Gondolesi, 2018, Argentina | No comparison between groups (see notes) | NR | NR | |
| Alhamad，2019, USA | NR | NR | NR | |
| Hau and Jahn,  2020,  Germany | SPK1/SPK2/KTA2:  AOD: 16.9/16.7/40  CHD: 25.8/50/73.1  Retinopathy: 87.6/66.7/38.5  Neuropathy:  73.3/75/46.2 | SPK1/SPK2/KTA2:  ALG/ATG:  70.8/91.7/15.4  IL2-RA:21.3/0/46.2  None:7.9/8.3/38.5 | SPK1/SPK2/KTA2:  MMF:80.9/91.7/84.6  SRL: 14.6/8.3/0  Multiple:3.4/0/0  None: 0.8/0/15.4 | |
| Fu, 2021,  China | KTA2/SPK2:  CVD: 23.7/28  CRD: 7.9/15.8  HBP: 94.7/97.4 | KTA2/SPK2:  ATG:7.9/97.4^@@^  IL2-RA:92.1/2.6^@@^ | KTA2/SPK2^@@^:  Tac:68.4/94.7  CsA:31.6/5.3  MMF:34.2/52.6  EC-MPS:65.8/44.7  Mizoribine:0/2.6 | |

Note: SPT1=T1DM recipients with SPK; SPT2= T2DM recipients with SPK; KTAT1= T1DM recipients undertaking deceased kidney transplantation alone; NR= not reported; CHD= coronary heart disease; AOD= Arterial obstructive disease; HBP= high blood pressure; CsA= cyclosporine A; EC-MPS, enteric-coated mycophenolate sodium; MMF,mycophenolate mofetil; AND= autonomic dysfunction; ATG= rabbit anti-thymocyte; ALG= anti-lymphocyte globulin; OKT3= muronab-anti CD3 antibody; ATG= rabbit anti-thymocyte globulin; IL2-RA= interleukin 2 receptor antibody (basiliximab/daclizumab); AP= antiproliferative; MMF= mycophenolate mofetil (includes nonenteric and enteric formulations); SRL= sirolimus; AZA= azathioprine; CAD= coronary artery disease; CVD= cerebrovascular disease; PVD= peripheral vascular disease; globulin; RTP=retinopathy; NRP= Neuropahty; *=there was no statistical significant between SPT1 and SPT2, **=there was statistical significant between SPT1 and SPT2, @=there was no statistical significant between SPT2 and KTAT2, @@=there was statistical significant between SPT2 and KTAT2. ***P < 0.05 for chi‐squared tests comparing differences between SPK,P+, SPK,P‐, DD‐KA, and LD‐KA groups; No comparison between groups: Retinopathy:78.2 Polyneuropathy:41.3; vasculopathy: 15.2; HBP:65.2; hypothyroidism:10.8; dyslipidemia:6.5; calciphylaxis:2.2; osteoporosis2.2; autoimmune hepatitis2.2)

Figure S1. Sensitivity analysis by excluding Fu et al.’s study in meta-analysis of 3-year pancreas survival rate after SPK


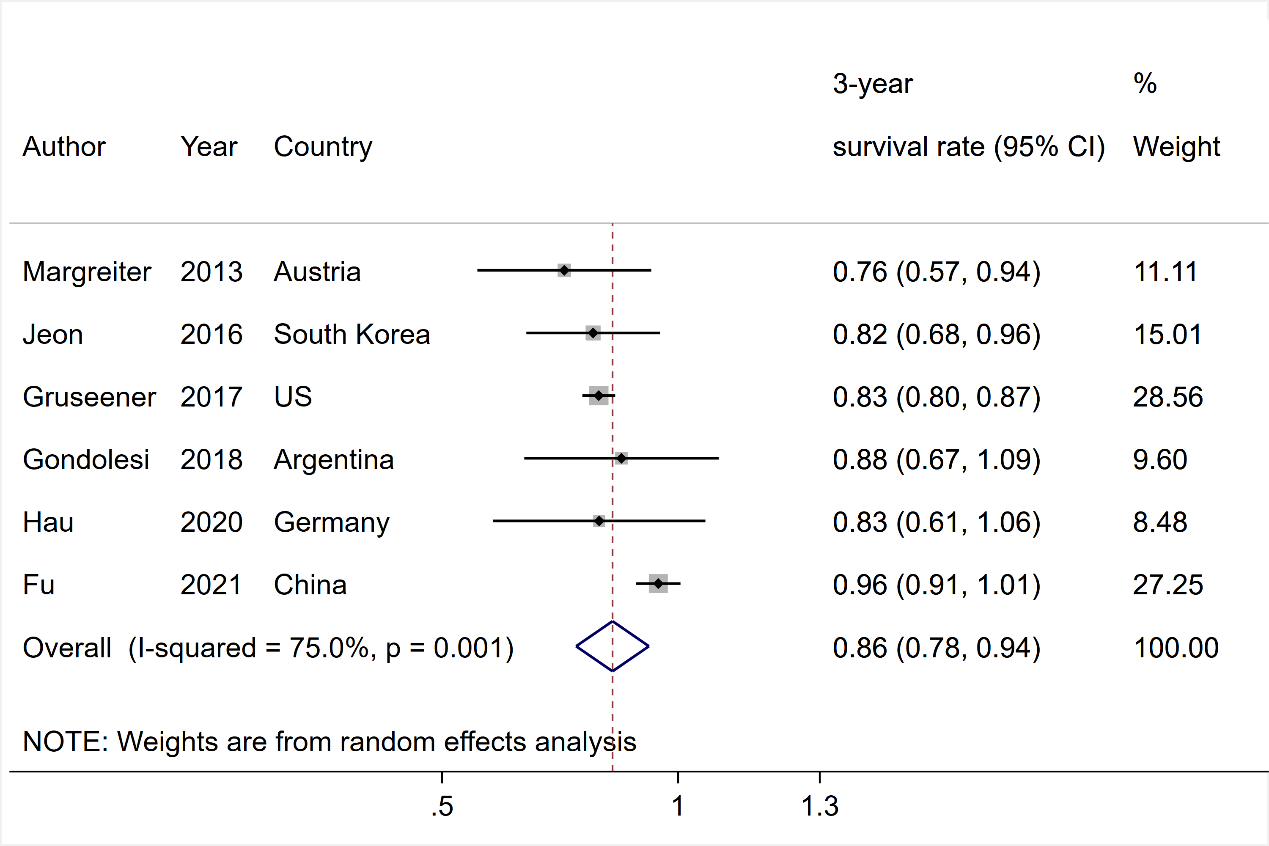


Figure S2: Forest plot for meta-analysis of risk ratio of complications betweenT1DM and T2DM


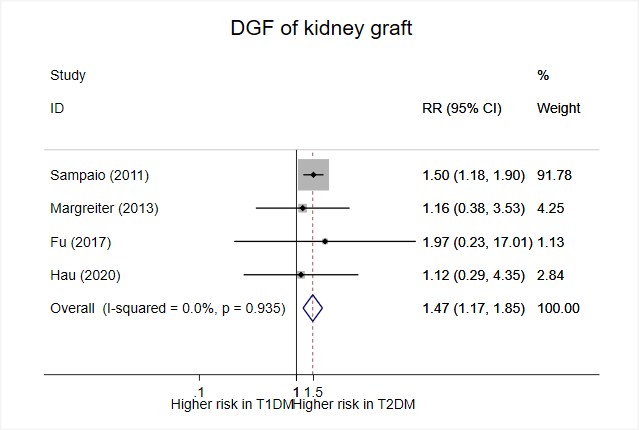

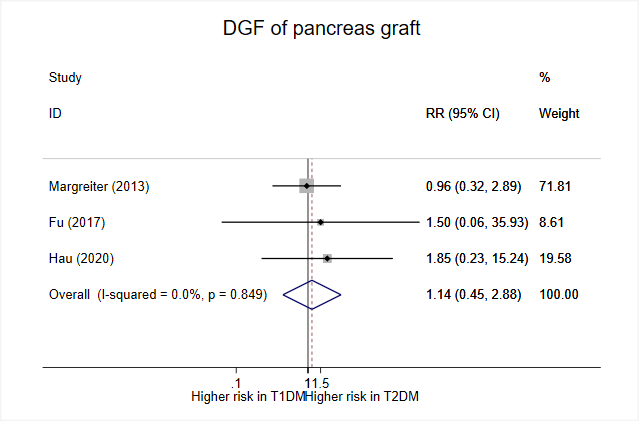


B

A


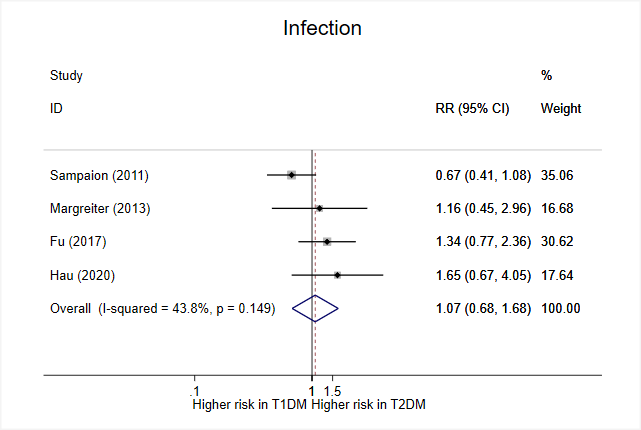

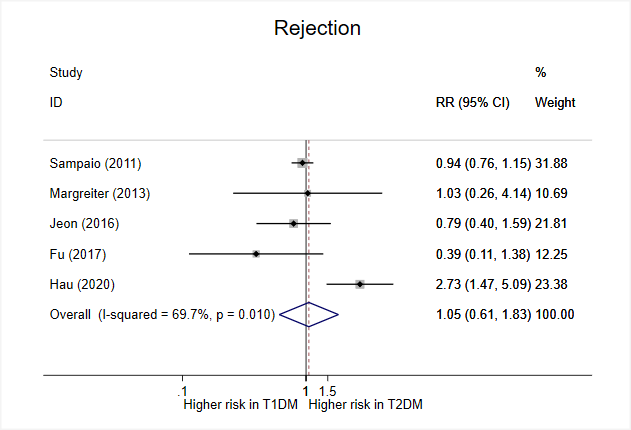


D

C

Figure S3. Sensitivity analysis by excluding Hau et al.’s study in meta-analysis of rejection risk of T2DM compared with T1DM


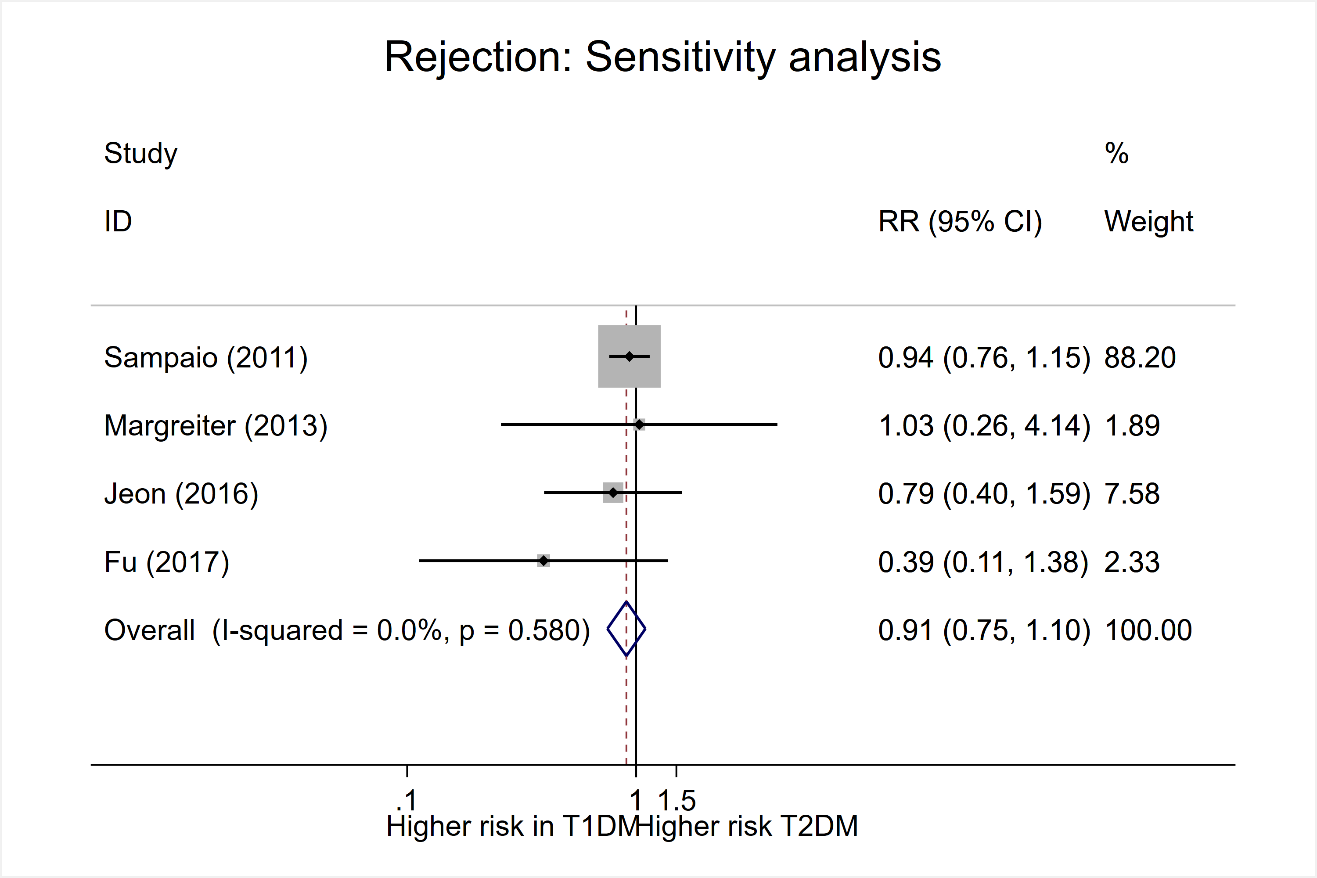


Figure S4. Forest plot for meta-analysis of risk ratio of complications between KTA and SPK


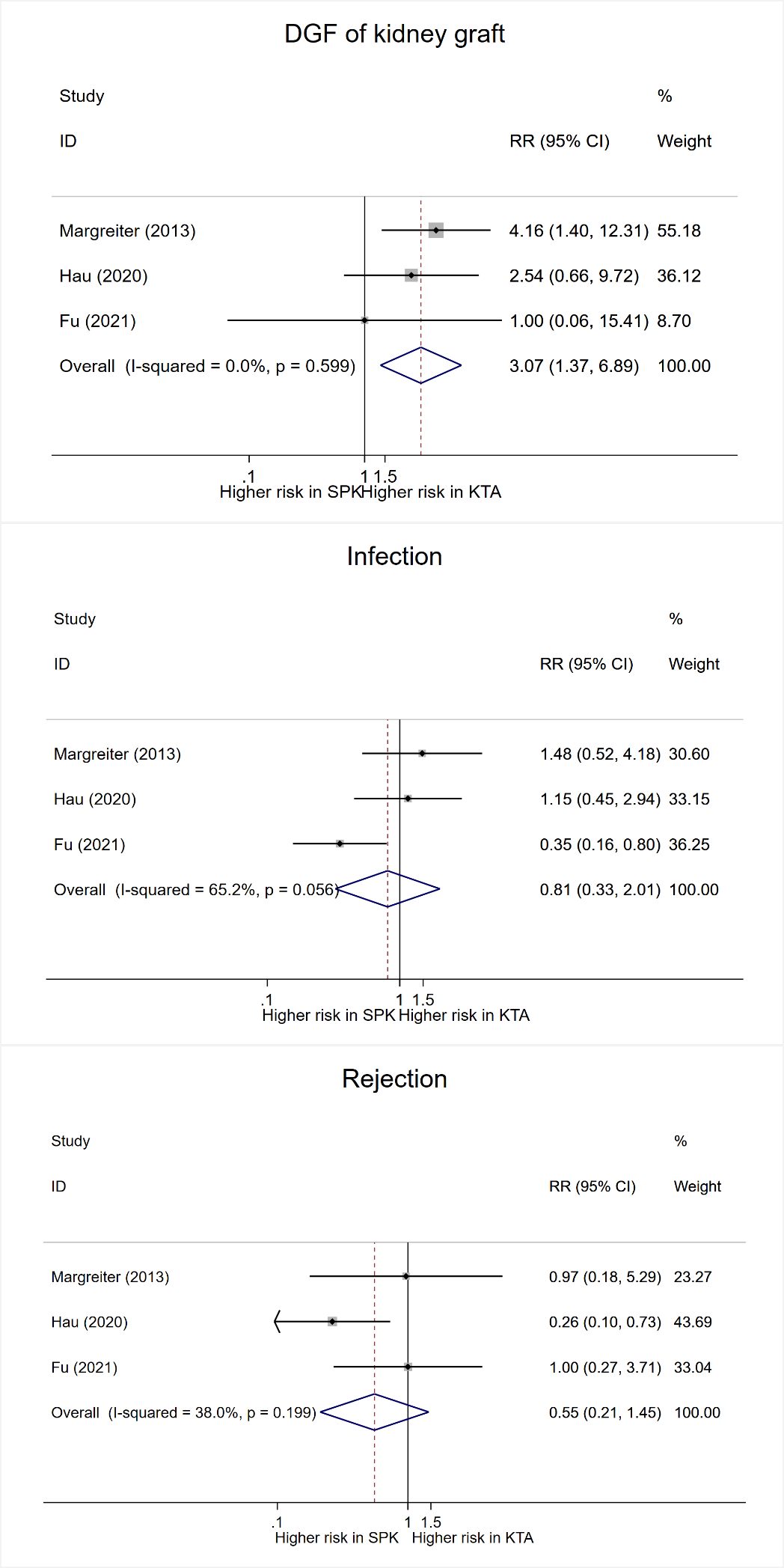


DGF of kidney graft
